# United Confederate Surgeon’s Association

**Published:** 1899-05

**Authors:** 


### United Confederate Surgeon's Association.

---

*Office of Surgeon General, United Confederate Veterans,*
*623 North Lafayette Square.*

NEW ORLEANS, LA., April 15, 1899.

*To the Survivors of the Medical Corps of the Army and Navy of the Confederate States.*

GENTLEMEN: The United Confederate Veterans will meet again in annual reunion, May 10, 11, 12, and 13, 1899. This will be our ninth reunion, and heroic and immortal Charleston, that world renowned and famous city of South Carolina, has been chosen for that great meeting of Confederate Veterans.

Surviving Comrades of the Medical Corps, you are urged and invited to come to that convocation of veterans as numerously as possible. But a few more years of usefulness remain to us, let us utilize them by promptly contributing, each one of us, our individual professional mite in valuable experience, for the historian to come. Bring with you, or send something in writing from the treasury of your own experience. Such contributions, addressed to me at Charleston, care of Major General George Moorman, Adjutant General and Chief of Staff, United Confederate Veterans, will reach me.

At the Atlanta, Georgia, Confederate reunion, held last year, conformably with my circular letter of June 30, 1898, mailed to all the then existing Camps of our Association, all the Surgeons and Assistant Surgeons reporting on that occasion, came together, framed and adopted a constitution and by-laws and organized under the same, by electing General Forest's, Distinguished Chief Surgeon, Dr. J. B. Cowan, of Tullahoma, Tennessee, as President, and the following celebrated Confederate Surgeons, Drs. J. McFadden Gaston, 1st Vice-President; R. C. Devine, 2d Vice-President, and J. J. Knott, Recording and Corresponding Secretary. I regret to say, that among our losses by death for the past year, 2d Vice-President Devine, has recently died, and at the moment when engaged in concluding a surgical operation. All but the President reside in Atlanta, Georgia.

At the approaching Charleston reunion, it will be in order to elect the officers of the United Confederate Surgeons' Association for the new year, dating from this approaching reunion, and to receive the report of work done during the past year.

It was a great pleasure to meet at the Atlanta reunion, that grand old veteran Surgeon of the Confederacy, Dr. S. H. Stout, who was the distinguished and only Medical Director of Hospitals of the Confederate Army and Department of Tennessee, which title also included all the territory protected by the said Army of Tennessee. Though full of years, his figure was erect, his step elastic, his eyes bright, and his intellect without the remotest semblance of a cloud.

He is the last surviving Medical director of our great Medical Corps, and is a great landmark to which we can all point with professional pride. And yet, recently, I have been called on to settle, adversely, the claim of another, a pretender, to the high office of Medical Director of the Confederate Hospitals above mentioned with territory also covered.

The submission of this important historical matter for my decision in the premises, came from a great Southern State, where resides now in quiet retirement the true and only Medical Director of the hospitals in question. This fact, this attempt to appropriate the high honors of another, challenges the importance of thorough organization, in order to preserve inviolate the reputations and the names of all the Surgeons and Assistant Surgeons who were faithful to the Southern Confederacy to the final surrender of her armies.

With the object of perfecting the roster of the Confederate Medical Corps, who served on sea and land, in field and hospital, I request from each now surviving Surgeon and Assistant Surgeon, the names of every Surgeon and Assistant Surgeon he or they can vouch for, who served faithfully to the end of the war between the States, together with the States from which such officers in question came, and such other information as may be appropriate to the purpose in view. Please mail this information to my New Orleans address.

And now, in conclusion, permit me to express the hope, that the Confederate reunion, soon to assemble at Charleston, will among other things be signalized by a very large attendance of the matchless Medical Corps of the Confederate Army and Navy, who with 50,000 more Federal prisoners under their care than Confederate prisoners in Federal prisons, lost 4000 less Federal prisoners, evidencing their superior skill under great and far reaching disadvantages.

Fraternally and sincerely your comrade,

C. H. TEBAULT, M. D.

Surgeon General United Confederate Veterans.

*Texas State Medical Association, Office of the Secretary.*

GALVESTON, TEXAS, May 5, 1899.

*To Officers and members of County and District Societies in Affiliation with the Texas State Medical Association.*

DEAR SIRS: I beg herewith to make the following announcement:

At the recent meeting at San Antonio, the report of the Committee on Society Organization was adopted, which contains these provisions, viz.: First, each county or district society is to pay to the Treasurer of the State Association annual dues of $5.00 for each twenty members or fraction thereof. My interpretation of this clause is, that there shall be an additional charge of $5.00 for each fraction over 10. Second, that the same rules will apply to societies as to members as regards payment of dues. Third, societies in affiliation are entitled to send representatives on the basis of membership, that is, one delegate for every 20 members or fraction thereof, who shall be exempt from payment of dues or initiation fees, and who must be members in good and regular standing in the societies they represent. My interpretation of this clause is that delegates will be entitled to the same privileges as members, inclusive of a copy of the Transactions, and that therefore, they will be members so long as they observe the rules applicable to other members. Every medical society in the State which adopts and conforms to the code of ethics of the American Medical Association, and which desires affiliation with the State Association upon the above mentioned basis, is requested to notify me at once, and furnish information upon the following points:

A. Number of members. B. Roster of officers. C. Place and date of meeting.

Annual dues should be sent to the Treasurer, Dr. R. F. Miller, Sherman, Texas.

Fraternally yours,

H. A. WEST, Secretary.

---

GALVESTON, TEXAS, May 6, 1899.

*Editor Texas Medical Journal.*

Please announce that the next semi-annual meeting of the South Texas Medical Association will be held in Cuero, on June 13th, prox., at 2 p. m. The meeting will be instructive and interesting, and all are cordially invited to attend.

O. S. HODGES, M. D. Secretary

H. A. WEST, M. D., President.

